# Nursing churn and turnover in Australian hospitals: nurses perceptions and suggestions for supportive strategies

**DOI:** 10.1186/1472-6955-13-11

**Published:** 2014-04-08

**Authors:** Angela J Dawson, Helen Stasa, Michael A Roche, Caroline S E Homer, Christine Duffield

**Affiliations:** 1 Centre for Midwifery, Child and Family Health, Faculty of Health, University of Technology, Sydney, Jones Street, Sydney 2007, NSW, Australia; 2 Centre for Health Services Management, Faculty of Health, University of Technology, Sydney, Jones Street, Sydney 2007, NSW, Australia

**Keywords:** Nursing staff, Hospital personnel, Turnover, Personnel management

## Abstract

**Background:**

This study aimed to reveal nurses’ experiences and perceptions of turnover in Australian hospitals and identify strategies to improve retention, performance and job satisfaction. Nursing turnover is a serious issue that can compromise patient safety, increase health care costs and impact on staff morale. A qualitative design was used to analyze responses from 362 nurses collected from a national survey of nurses from medical and surgical nursing units across 3 Australian States/Territories.

**Method:**

A qualitative design was used to analyze responses from 362 nurses collected from a national survey of nurses from medical and surgical nursing units across 3 Australian States/Territories.

**Results:**

Key factors affecting nursing turnover were limited career opportunities; poor support; a lack of recognition; and negative staff attitudes. The nursing working environment is characterised by inappropriate skill-mix and inadequate patient-staff ratios; a lack of overseas qualified nurses with appropriate skills; low involvement in decision-making processes; and increased patient demands. These issues impacted upon heavy workloads and stress levels with nurses feeling undervalued and disempowered. Nurses described supportive strategies: improving performance appraisals, responsive preceptorship and flexible employment options.

**Conclusion:**

Nursing turnover is influenced by the experiences of nurses. Positive steps can be made towards improving workplace conditions and ensuring nurse retention. Improving performance management and work design are strategies that nurse managers could harness to reduce turnover.

## Background

High rates of nursing turnover where staff voluntarily leave or transfer from their primary employment position to another position in nursing, or to another profession [1] (described as between 12% [2] to greater than 50% turnover [3]) are a problem currently affecting many countries [1,4,5]. In Australia, few studies have examined nursing turnover rates [6,7], however recent reports indicate a turnover rate in one state (New South Wales) of 1 per cent to 1.4 per cent per month [8,9]. A term used to define significant turnover “churn”, is defined as involuntary staff movement, where nurses are moved to other positions or locations within the organization. It is also important to consider this aspect of turnover also as such staff movement can negatively impact upon skill-mix, scheduling and continuity of care [10].

Minimising turnover rates is an important priority for health service managers for a number of reasons. Firstly, turnover is costly for health care organizations with a pilot study in Australia estimating turnover costs at $A16,634 per nurse [1]. Secondly, turnover affects the roles [11], morale and stress levels of remaining staff, impacting upon nurse productivity [1,12]. Finally, turnover impacts on patient safety and outcomes with registered nurse (RN) turnover found to be related to both increased infection and subsequent hospitalization [13]; an increased likelihood of medical error [1,14]; and reduced patient satisfaction [15].

### Factors contributing to nursing turnover

In order to reduce the rate of turnover, it is necessary to identify exactly which factors contribute to this phenomenon. A number of descriptive studies from different countries have used surveys to examine some of the reasons for nurse turnover [6,14,16]. However the mechanisms that underpin nurses’ decisions to leave are not well understood [17].

In the international literature, the work environment has been identified as one important factor in nurse turnover. For example, Aiken and colleagues in their research in the US, Canada, England, Scotland and Germany, found that low morale, management issues, workload, and the amount of time spent on non-nursing tasks all contributed to turnover [18]. In Canada, Leiter and Maslach [19] found that burnout or exhaustion mediated the occurrence of turnover. Another study by Rhéaume and colleagues [20] found that a key component of the work environment, foundations for quality nursing care, was the top predictor of the variance in turnover. In the US, Johnson and Rea [21] found that workplace bullying was associated with turnover from both the organization and the nursing profession. Finally, in Taiwan, Chen and co-workers found that distributive justice, workload, resource adequacy, supervisory/kinship support, and job satisfaction were strongly associated with intention to stay or leave one’s job [22].

Yet, whilst factors related to the work environment are important contributors to nurse turnover, such research tends to focus on the behaviour of the “average nurse” [23]. This is problematic, because it is possible that individual differences between nurses and groups of nurses are also influential in turnover [23,24]. For instance, one Australian study found that for some nurses individual affective professional commitment was significantly related to intention to change professions [25]. Other individual factors which may play a role in turnover are generational membership and age [26,27] or years of experience [4].

### Reducing nursing turnover

Of the research which has examined ways to reduce nursing turnover, Gess and colleagues found that changing organizational processes, so that the nursing staff had additional autonomy and were offered rewards and recognition for their work improved organizational commitment, and decreased turnover [28]. In another study, Porter and co-workers instituted a nursing labour management partnership (NLMP) program, which encouraged a collaborative approach between management and the nursing union [29]. This collaborative approach was found to improve satisfaction and reduce turnover.

There is little Australian research on the causes of nurse turnover. Additionally, of the research which has been done, most of it has been in the form of survey data, or quantitative analysis, rather than qualitative studies [30]. This could result in a limited understanding of factors associated with nurse turnover. Whilst quantitative data can provide a broad snapshot of the findings, survey data usually lacks the rich insight needed to gain a better understanding of the causes of turnover. Qualitative research provides a deeper understanding and insight into the everyday realities of the nursing work environment from the perspectives of nurses themselves.

### Purpose of the study

This study adds to the small body of qualitative knowledge into experiences of nurse turnover in public hospitals [31,32], and aims to provide insight into;

• nurses’ perceptions of the working environment on medical surgical wards;

• factors contributing to nursing turnover from the perspective of nurses in hospitals in Australian states and territories; and

• possible strategies to improve working environments and improve retention.

This study therefore sought to ask “What factors in the work environment do nurses themselves believe are related to turnover, and what retention strategies do they propose?”

## Methods

A descriptive qualitative study was undertaken to explore the perceptions and views of Australian nurses of the factors that affect turnover and the working environment [33].

Ethical approval was sought from and granted by seven Human Research Ethics Committees representing the participating hospitals: Australian Capital Territory Health and Community Care Human Research Ethics Committee (The Canberra Hospital); Calvary Health Care ACT Human Research Ethics Committee (Calvary Hospital); Central Coast Area Health Service Ethics Committee (Wyong Hospital, Hornsby-Kuringai Hospital); Northern Sydney Health Hawkesbury Human Research Ethics Committee (Royal North Shore Hospital); Western Australia Country Health Service Board Research Ethics Committee (Armadale-Kelmscott Memorial Hospital); Department of Health Western Australia Human Research Ethics Committee (Sir Charles Gairdner Hospital, Royal Perth Hospital, Fremantle Hospital, Bentley Hospital; Osborne Park Hospital, Swan-Kalamunda Hospital). Ethical approval was also gained from the University of Technology Sydney Human Research Ethics Committee.

### Study participants

Participants were part of a broader study, which examined the relationship between nurse turnover and patient, organizational and staff outcomes. Patient, nurse and costing data were collected on 62 medical and surgical nursing units in 11 public hospitals across two states and one territory of Australia. Each nursing unit had two data collection periods, spaced 12 months apart, over a two-year period (2008-2010). Participants were registered nurses (RNs; Bachelor degree level), enrolled nurses (ENs; Diploma level), medication endorsed enrolled nurses (EENs; Diploma plus medication administration course) and assistant in nursing (AIN; Vocational Certificate). All RNs, ENs, and AINs engaged in clinical practice on sampled units were asked to complete a survey that gathered information about the practice environment, job satisfaction, physical and mental health, nurse leadership and demographics. Nurses’ experiences and needs on the subject of nursing turnover were sought at the end of the survey where an open question followed by a blank A4 page was given for participants to write their responses. The open question asked “Please provide any additional comments you would like to convey”. A total of 1655 nurses provided written consent and completed the survey, a response rate of 44.4%. While 21.8% (n = 362) of those who responded chose to add comments, personal reflections and narratives at the end of the survey. These data formed the basis of the analysis.

### Data analysis

The handwritten responses were entered into Excel and then imported into NVivo, a qualitative data analysis software tool. These were analyzed thematically [34] using an inductive process to categorise, tabulate and recombine the evidence, in line with the aim of the study reported in this paper. Words or short phrases were assigned codes, or terms that best captured the meaning of the participants’ expressions. Codes emerged during several readings of the responses and labels were assigned based upon the text and revised accordingly as new data were analysed. Codes were then grouped into categories and concept maps were drawn to identify relationships across all categories and sub categories which assisted to identify themes. The rigour of the analysis process was ensured through ongoing discussion between the researchers, who agreed upon categories and emergent themes.

## Results

Handwritten responses from 362 nurses were gathered and analysed. Table 1 provides an overview of the demographic data. It can be seen that respondents were mostly female, 40 years and over with a mean of nearly 14 years nursing experience. The participant characteristics in this qualitative study show similarities with the entire survey sample. However 63% of the responses in the qualitative study were from RNs compared to 80% of RN the responses in the survey sample.

**Table 1:** Characteristics of nurses who contributed qualitative responses to the turnover survey

	**State 1**	**State 2**	**State 3**	**Overall**
	**Mean (SD)**	**Mean (SD)**	**Mean (SD)**	**Mean (SD)**
**Age**	39.9 (11.57)	44 (11.34)	42.0 (12.18)	41.70 (11.91)
**Years nursing**	12.7 (10.77)	13.9 (11.62)	14.5 (11.98)	13.9 (11.56)
	**N (%)**	**N (%)**	**N (%)**	**N (%)**
**Gender**				
Female	100 (91%)	50 (83%)	172 (90%)	322 (89%)
Male	10 (9%)	10 (17%)	20 (10%)	40 (11%)
**Employment status**				
Full time	57 (52%)	40 (67%)	106 (55%)	203 (56%)
Part time	35 (32%)	20 (33%)	73 (38%)	128 (35%)
Casual*	18 (16%)	0 (0%)	13 (7%)	31 (9%)
**Grade**				
RN	63 (57%)	36 (60%)	129 (67%)	228 (63%)
EN	23 (21%)	22 (37%)	24 (13%)	69 (19%)
AIN	2 (2%)	0 (0%)	4 (2%)	6 (2%)
**Other	22 (20%)	2 (3%)	35 (18%)	59 (16%)
**Overall**	**110 (30%)**	**60 (17%)**	**192 (53%)**	**362**

*Casual employment refers to working only on demand by the employer, typically as permanent part-time employees.

**Includes educators and managers.

**Figure 1:**
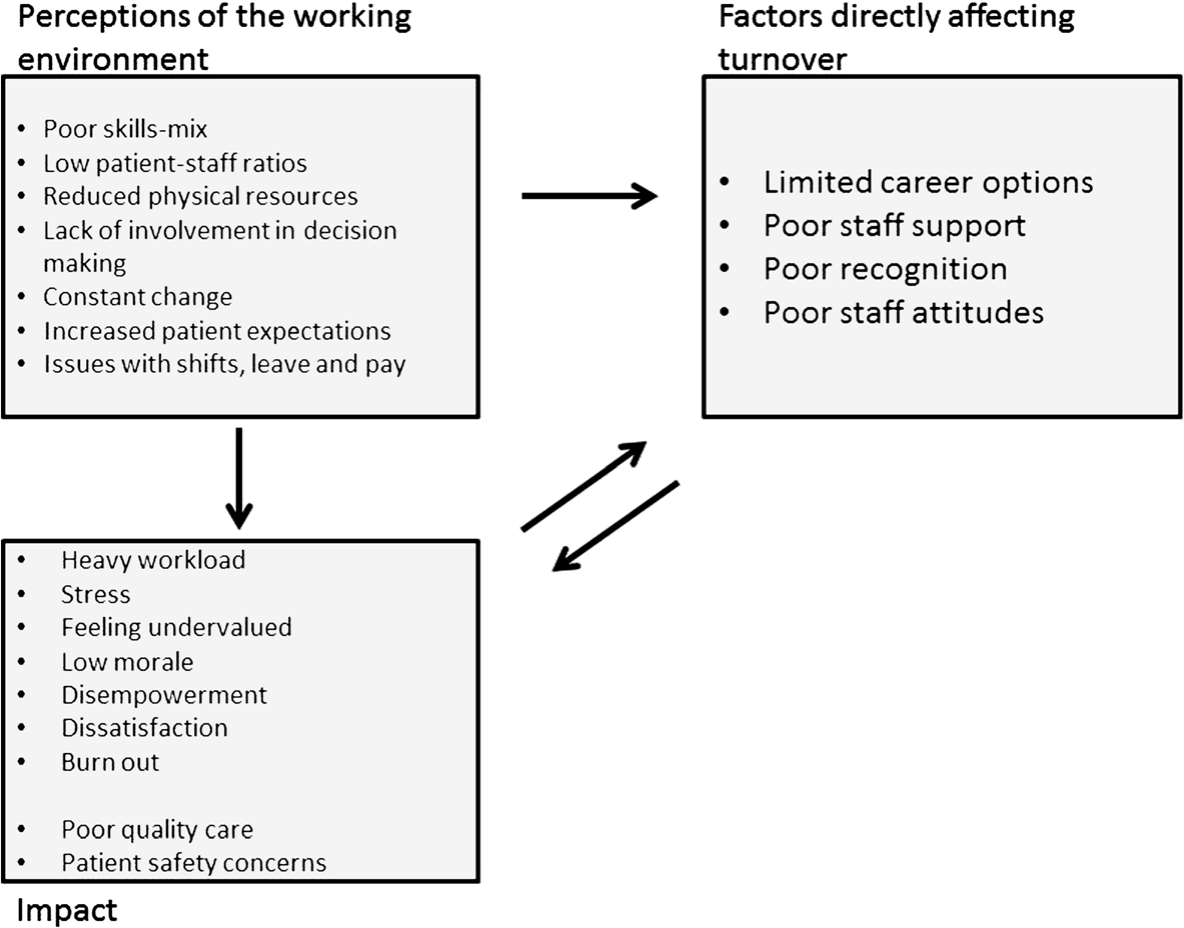
Relationship between factors reported to affect the nursing working environment and their impact upon nurses and their decision to remain in their jobs and the profession.

Key themes identified are described below according to the study focus on the nurses’ perceptions of: 1) their working environment and its impact on them; 2) factors directly related to retention; and 3) strategies to help to reduce turnover and improve the working environment. The relationship between these three themes is shown at Figure 1.

### “A poor work environment is the major obstacle to enjoying my role”

A poor work environment included concerns about the skill-mix involving different levels of workers across the team at any particular time; the low nurse:patient ratios; a lack of involvement in decision making; dealing with constant change; issues with shift work; leave and pay; and increased patient expectations. These will be examined in turn.

#### *Poor skill-mix*

Poor skill-mix was commonly cited as a serious problem at work. This was due to a large number of new graduate nurses; a large casualised workforce (agency [contract] and hospital pool staff) with different levels of experience; high use of EENs to undertake tasks that required RNs to supervise closely; and large numbers of nurses with overseas training, who although registered, were regarded by Australian qualified nurses as lacking skills. Nursing students were also described as being used as “staff” which was considered inappropriate (nursing students are formally considered ‘supernumerary’ in the Australian context). These skill-mix issues were said to increase workload, and meant nurses were not able to complete shifts on time and increased stress (by placing unrealistic expectations and demands on qualified staff). One respondent wrote:

Recently we have had an increase in new graduate Nurses and employed more overseas Nurses - This has reduced our expertise, skill mix and caused our greatest concerns these last 2 months. RN, 48 years, Part time Female.

The inclusion of large numbers of overseas qualified nurses was described as particularly problematic as variation in skills levels not only increased the workload of other staff, but language differences led to communication problems that could be dangerous in emergency situations.

*Nurses from NESB* [Non English Speaking Background] *who are very difficult to understand due to strong accents, resulting in communication problems and errors. RN, 47 years, Full time, Female.*

Overseas qualified nurses themselves described discrimination resulting from the way they communicated and uncertain employment prospects.

#### *Low nurse-to-patient ratios*

Despite increases in particular cadres and groups of staff, nurses said that nurse-patient ratios were often low as a result of absence due to injury and illness and where budget cuts had reduced staff supply. Many nurses commented that nurse-patient ratios were inappropriate and that management was unresponsive when they reported shortages. One participant wrote:

[I] Feel nurses are overworked and higher management know this. We are expected to deliver the same care and same admissions, whether we have adequate nurses on or not. We are expected to ‘special’ (provide one to one care) patients, whether we are given the staff to do this or not. “Absorb” them we are told- how do you this? EN, 50 years, Part time Female.

Staff shortages were said to lead to low levels of job satisfaction and increase nurse workloads, absenteeism and workplace injuries. In addition, staff shortages were reported to delay patient treatment and increase health care costs.

#### *Lack of involvement in decision making*

Many of the comments described situations where nurses felt left out of unit management decision making processes and they felt particularly aggrieved when they had solutions to address the issues. Nurses described feeling undervalued and disempowered by management or by the attitudes of doctors. For example:

*Upper Hospital management i.e. above ward level, seem to enforce different work policies that impact greatly on RNs who work as hands-on practitioners. Their decisions are made without consultation with nurses and NUMs* [Nurse Unit Managers]. *It’s these decisions that make delivering a high standard of nursing care consistently very difficult. Educator/Manager/Other, 45 years, Part time Female.*

There were also statements indicating that a lack of involvement in decision making devalued nurse’s contributions to the extent that they felt there was no point continuing to practice.

*No support. Nurses require more autonomy decision making and real input into patient care* & *treatment. Intention to leave to a different profession once requalified. There is no incentive to stay whatsoever. RN, 23 years, Full time Female*.

#### *Issues with shift work, leave and pay*

Low pay, inflexible shifts and limited leave allowance were commonly cited as being demotivating, disruptive and stressful. Nurses felt management were not supportive in helping them to achieve a life-work balance. One nurse wrote:

Mature age nurses [are] not being given choices (i.e. wanting to take less senior role i.e. as in coordinator of shift). [There is] unfriendly rostering! (i.e. late shift, day off, then early shift quite often). RN, 54 years, Part time, Female.

In some cases, hospital employment policies that focused on the casualization of staff were regarded as contributing to inefficiency and disruptions.

#### *Increased patient expectations*

Nurses described patient expectations as having increased over the years due to higher knowledge levels. Meeting these expectations in conditions of stress, reduced staff and constant change was difficult. Managing family expectations was equally challenging as described by this respondent:

Patients are sicker and older and frequently ruder; relatives are more demanding and rude. Educator/Manager/Other, 44 years, Full time, Female.

### “I’ve seen dramatic changes involving nurse’s turnover”

Respondents wrote about issues that led them to contemplate leaving the workplace. These included perceiving that there were limited career and employment choices; poor management support and little recognition; and a poor workplace culture and negative staff attitudes.

#### *Limited career and employment options*

Nurses provided numerous descriptions of experiences that had led them to contemplate resigning. These included situations where requests for transfers to other wards or positions was not available or had been denied, or where positions had been withdrawn despite the respondent being selected and appointed. For example:

The opportunity to transfer between hospitals due to relocation is non-existent. That is the reason I am searching for work within another industry currently. AIN, 23 years, Full time, Female.

Nurses indicated that the shortage of career opportunities and even difficulty finding a job as a new graduate nurse was leading them to contemplate other occupations. Graduate nurses were also said to get limited clinical experience before graduation, which reduced their ability to progress in their current profession and resulted in their looking for new careers. One nurse wrote:

I see grad [graduate] nurses struggle in their first 12 months in the hospital setting as they don’t get enough clinical practice and most hospital trained nurses agree! These nurses eventually leave after 12 months and seek new careers. E.g. teaching. RN, 58 years, Full time, Female.

#### *Poor staff attitudes and workplace culture*

Poor staff attitudes were said to affect workplace culture leading to a break down in relationships. The negative workplace culture prompted some staff to leave and seek other jobs. For example:

Who in their right mind would want to be a nurse? Nursing recruitment and retention is only going to get worse in the next 10-20 years because our culture now breeds selfish individuals instead of caring people who see others as part of their families. RN, 56 years, Full time, Male.

According to one nurse, this workplace culture had reduced the nursing role to one that is unskilled and disempowering. These very negative reactions were typified in this response:

Don’t get me started on tyrant bosses, …… and horizontal violence from other nurses. Such a boring subject! In some other cultures, nurses administer medications and deliver technical care, while families do the basic nursing care and feeding - that will never happen here, nursing a very sad game. RN, 46 years, Full time, Female.

Poor relationships with staff and patients were said to lead to stress, burn out and turnover that ultimately reduced patient care. These experiences led nurses to plan to leave, thus contributing to future turnover, for example:

I plan on leaving nursing within the next year. Nursing has become physically heavy. Patients constantly complain about poor service. Management don’t work well or appreciate staff. Doctors treat nurses as if they are inferior. Nurses leave this job stressed and burnt out and likely physically injured. RN, 32 years, Part time, Female.

### “Improvements and much more encouragement is required”

A number of improvements were suggested. These included the provision of employment options, rewarding good performance, enhancing professional development and training opportunities and improving management practices.

#### *Providing employment options*

The availability of a range of employment options, including the provision of secondments or temporary transfers to another position, annual leave choices, casual employment and part time employment increased nurses’ job satisfaction. These options enabled nurses to achieve family-friendly hours; take time off to attend to personal issues and family responsibilities; gain additional professional experience; and attain a suitable level of flexibility and variety to maintain interest. One nurse wrote:

*There are only three reasons that I remain working on this ward. 1) I can work night duty only which fits in with the care of my preschool age children 2) I can work part time and get rostered the shifts that I mostly want re night duty ×2/week, split shifts (at my request as this works for me) however I have to fight for this 3) the “atmosphere” with the night nurses is good. RN, 37 years, Part time, Female*.

Nurses commented on the need to increase opportunities for casual employment and night shifts, as they felt that such work could provide some relief from stress. For example:

I stay on [the] casual pool and move around the hospital. This reduces the stress a bit in my interactions with staff and management. RN, 30 years, Casual, Female.

Yet, conversely, for other nurses, job satisfaction was linked to stable and regular shifts. Where this was not the norm, one nurse recommended a review of employment policies to address the disruption caused by constant change. She/he wrote:

I work on a casual basis. Therefore, I’m always sent to different wards. This is a disadvantage to myself and the staff on each ward. I used to work on Spinal Unit. I will pick up shifts where I can on that ward. When I returned from overseas, I tried to go back to spinal but “management” wouldn’t sign me off on the paperwork to allow my NUM to employ me. This is ridiculous, because they would have been saving themselves money. “Management” need to re-evaluate their employing policies. EN, 27 years, Casual, Female.

#### *Rewarding performance*

Performance review was seen as a way to recognise high performance although more attention to recognise achievements, encourage staff and reward excellent service was needed. One respondent wrote:

More focus should be on appraisals and recognition from the above (administration) as it is a hard job and we all have stress from personal issues too. RN, 42 years, Full time, Female.

Nurses who said that they were satisfied with their jobs noted that their current employment provided them with the space to appraise their strengths and consider their future personal as well as professional aspirations. For example:

I would like to be a better nurse! But it seems to me that I’ll only a better nurse if I become a better person. Nursing has given me the opportunity to do this, and I’m free to go and meditate because of the money I earn while nursing. So it’s a win win kind of thing really. Clinically I’m ok. I’d rate my ability at about 6-7 out of 10. I can handle most situations (clinically). BUT would like to study and work in the intensive care unit (ICU) maybe one day. RN, 38 years, Full time, Male.

#### *Enhancing professional development and training*

A common sub-theme was the need for quality preceptorship (supervised clinical practice) on the ward and professional development opportunities particularly for new graduates and overseas qualified nurses. One respondent wrote:

Need more staff development nurses on hand so we can continue to give our patients the care they require or someone to watch and guide nurses in training and give team time decrease our stress load. RN, 54 years, Part time, Female.

However, many nurses felt that major improvements needed to be made in the quality of clinical educators, including appraising their performance and reducing their administrative duties.

It would be a benefit to have educators working with nurses on the “floor” and appraisals to be attended on effectiveness of educators by the nurses working in the units. RN, 49 years, Full time, Female.

In addition, a number of nurses suggested improved pre-work orientation for nurses qualified overseas before they took up positions in Australia.

#### *Improving management practice*

Nurses described management changes they believed were needed to improve the working environment. This includes engaging with staff to improve decision making processes and being more responsive to needs. In addition, nurses felt that managers could improve the working environment and staff relationships through modelling behaviour and aligning leadership approaches. For example:

Senior and management nurses should also strive to “set the example” by both their own behaviour and interaction with nursing staff in all positions. Educator/Manager/Other, 53 years, Full time, Female.

There was also a need for managers to clarify roles and responsibilities so that guidelines and procedures could be better followed to improve performance:

Guidelines would work better if medical + nursing staff were each aware of their responsibilities. RN, 37 years, Full time, Female.

## Discussion

Our study found that nurses working in hospital wards in Australia consider limited career opportunities, poor staff support, recognition and attitudes of co-workers to be particularly influential in deciding to remain in their current job and the profession. This was linked to poor work environments characterized by inadequate skill-mix, low nurse-to-patient ratios and reduced physical resources, accompanied by a lack of involvement in decision making, constant changes, issues related to leave and shifts, poor staff relationships and patient expectations.

A number of suggestions were made to encourage retention. Interestingly, these focused on non-financial incentives emphasizing the recognition of staff achievements, the provision of appropriate career and professional development opportunities, and the need to clarify roles, engage with staff and model leadership. Nurses also highlighted the need for flexible employment options.

These self-reported factors are consistent with the literature. The importance of recognizing staff achievement is identified in studies in several countries where offering praise for a job well done was found to contribute to staff satisfaction [7,35,36]. Other research has found that strategies suggested by the nurses in our study have made an impact upon retention. Hunter and Nicol [37] found evidence that training and development, continued education, and professional growth opportunities improve recruitment and retention for occupational therapists. In our study, a lack of support for staff was regarded as a factor that contributed to nursing turnover, and participants highlighted the need for quality preceptorship for new graduates. Chenoweth et al. [38] have found that effective clinical supervision had a positive influence on recruitment and retention and worked best when there was a good supervisor-mentee relationship. In terms of the flexible working options requested by nurses in our study, other research has found that options such as self-scheduling system, flexibility in schedules, family-friendly policies and social hours improve health care provider retention [37-39]. Patterson et al. [40] also reported a positive influence between nurse staffing models and turnover although others have not found any evidence to suggest that having the right staff skill mix is effective in retaining health care providers.

### Implications for nursing management

A range of interventions have been found to address potential turnover and improve retention. The recognition of staff achievements and improving self-efficacy and participation in the workplace could be realized through improved performance management and flexible work design particularly in this Australian hospital context.

#### *Performance management*

Performance management (PM) for nursing staff may deserve focus as it provides processes for joint review that enable nurse and supervisor to plan for professional development and career advancement, recognise and reward staff achievements [41,42] and improve morale [43]. PM processes that provide such opportunities may improve job satisfaction and retention [42,44] and help to address factors that contribute to nursing turnover. PM enables the achievement of accomplishment-based performance indicators to be negotiated in line with nurse’s job descriptions, standards and outcomes at the individual team and hospital levels. This process should engage staff members themselves in appraising their performance and planning and evaluating appropriate career and professional development activities [45,46]. Enhancing PM systems alongside building nurse manager-nurse relationships can also provide the support and mentoring that participants in our study described as necessary to plan and achieve promotion. Additionally, managers offering praise when high quality clinical practice is observed may help to reinforce the occurrence of such practice, and ensure that nursing staff feel that their work is appreciated and valued.

Nurses in our study also identified a need for clinical preceptorship for overseas qualified nurses and new graduates, the latter group being of particular concern in relation to turnover in Australia [47] and overseas [20,48]. The difficulties of overseas qualified nurses in Australia noted in our study have been found in other research [49,50]. A PM process could also include appraisal of cultural competency [51] and identify professional development interventions to address cultural and language issues [52-54]. In this way, early identification of learning needs may enable managers to ensure that overseas qualified nurses and new graduates can receive appropriate training before taking on shifts in the ward.

#### *Work design*

In our study participants highlighted poor skill-mix and issues with shifts that could be addressed by the provision of a range of employment options and flexible shifts. Flexible scheduling has been found to be an important part of a quality work environment because nurse satisfaction is connected with having a certain level of influence on decisions pertaining to the hours they work [55]. However, challenges to flexibility include nurse shortages and complex staffing arrangements. Nonetheless, Hirschkorn and others [56] have identified lessons which could potentially be applied to the nursing context such as flexible work environments and phased retirement options, which may benefit working parents and carers as well as an aging workforce.

### Role of nursing unit manager and nursing executive

The effective leadership of the nursing unit managers (NUMs) is critical to improving staff retention and reducing turnover, as well as improving nurse satisfaction and the provision of a positive a working environment [7]. However, building the leadership capacity of NUMs in order to strengthen performance management and introduce flexible work design tailored to address the different context of hospital wards [57] requires organisational support from nursing executives. This nursing executive must itself be stable and have opportunities for growth within its mandate [58]. The recommendations of the public enquiry into the Mid Staffordshire Trust in the UK provides clear evidence of the importance of nursing leadership and management in creating positive work environments, recognising nurses’ contributions and addressing morale [59].

### Limitations

There are a number of limitations that may affect the interpretation and transferability of the findings of this study. The response rate in this qualitative study (n = 362) reflects 22% of the total population (n = 1655) included in the larger survey. There may be the possibility that participants who answered the open-ended question were not typical of many nurses’ perspectives which may limit data quality. The findings reflect the voices of nurses who were highly motivated to contribute their views. However those that chose not to complete the long answer question may not have wanted to add additional material, did not have the time or may have not been confident to share their feelings and experiences. Member checking to confirm understanding and ensure that the themes adequately represented the expressions of the participants was not possible due to the fact that the surveys were completed anonymously making to follow up difficult. In addition the written response does not allow for further probing or clarification of responses as one would be able to achieve in a face to face interview.

## Conclusion

Positive solutions must be found and nurses engaged in designing, implementing and evaluating them if we wish to decrease clinical nursing staff turnover. Enhancing performance management and flexible employment options require changes in workplace culture to ensure that nurses don’t view nursing as a “dead end job”. Nurses need to feel empowered to help steer their career and contribute to decisions that progress the delivery of quality health care on the hospital wards in which they work.

## Competing interests

The authors declare that they have no competing interests.

## Authors’ contributions

All authors contributed to the drafting of the manuscript, HS undertook the literature review and AD drafted the manuscript. CD and MR designed and co-ordinated the study. AD led the analysis and all authors critically discussed each step. The final manuscript was read and approved by all authors.

## Pre-publication history

The pre-publication history for this paper can be accessed here:

http://www.biomedcentral.com/1472-6955/13/11/prepub
